# Design and Simulation of a High-Efficiency Tunable Terahertz Absorber Based on Patterned Graphene

**DOI:** 10.3390/nano16160998

**Published:** 2026-08-13

**Authors:** Shuai Wang, Liang Xu, Qingfeng Yang, Yan Xu, Haiyun Yao

**Affiliations:** 1 School of Opto-Electronic Engineering, Zaozhuang University, Zaozhuang 277160, China; beryly@shu.edu.cn (S.W.); 261909921@qq.com (L.X.); 2 Library, Zaozhuang University, Zaozhuang 277160, China; 254604980@qq.com

**Keywords:** THz absorber, graphene, tunable absorption bandwidth, polarization insensitivity

## Abstract

The rapid expansion of terahertz (THz) communication, nondestructive testing and biosensing puts forward urgent demands for absorbers with switchable working bandwidth, yet parts of existing graphene-based absorbers adopt costly noble-metal backplanes and can hardly realize reversible narrow and broadband absorption conversion. In this work, a three-layer metamaterial absorber is designed, where low-cost tungsten replaces precious metals as reflective substrate, polyimide serves as intermediate dielectric and patterned graphene composes the top absorbing layer. The finite element method (FEM) is employed to investigate the synergistic modulation of THz absorption characteristics by graphene’s Fermi level (*E_f_*) and relaxation time (*τ*) across the 0–6 THz frequency range. Simulation results reveal that increasing *E_f_* from 0.1 eV to 0.9 eV effectively broadens the effective absorption range. At fixed *E_f_* = 0.9 eV, dual discrete absorption peaks with peak absorptivity up to 99.8% emerge at *τ* = 0.1 ps, while reducing *τ* to 0.05 ps enables an ultrawide 2.5 THz high-efficiency absorption band (absorptivity ≥ 90%) including a 1.4 THz near-perfect absorption (absorptivity ≥ 99%) region. Benefiting from high geometric symmetry, the proposed structure exhibits polarization-insensitive absorption and stable performance for incident angles up to 60°. This numerical work provides design references for low-cost switchable THz absorbers.

## 1. Introduction

Terahertz (THz) waves (0.1–10 THz [1]) occupy a distinctive electromagnetic spectrum region between microwaves and infrared light, bridging macroscopic electronics and microscopic photonics. Benefiting from their intrinsic properties including low photon energy, strong penetration, and fingerprint spectral characteristics, they show great application potential in 6G communication [2], security screening [3], biomedical diagnosis [4], and material characterization [5], and have attracted extensive research interest worldwide. However, THz technology industrialization is bottlenecked by the lack of high-performance functional devices. Among them, THz absorbers are the key for wave manipulation and energy conversion, determining THz systems’ accuracy, efficiency, and anti-interference capability [6]. Most conventional THz absorbers adopt a metal–insultor–metal (MIM) sandwich structure. Restricted by the inherent electromagnetic properties of constituent materials, they generally support only a small number of resonant modes, resulting in single-frequency or narrowband absorption performance [7]. To address this limitation, metamaterials have attracted widespread attention in electromagnetics research. These artificially engineered materials enable flexible and versatile control over the amplitude, phase, polarization, and propagation of THz waves, providing an effective approach for the realization of THz functional devices [8]. However, in practice, metamaterial absorbers still suffer from inherent drawbacks that need to be addressed, including the difficulty in regulating their electromagnetic or optical properties, which prevents them from meeting the requirements for tunability. Notably, graphene, a two-dimensional Dirac material, possesses high carrier mobility and electrically tunable Fermi level (*E_f_*), making it a promising candidate for tunable THz devices [9]. In addition to the electrically tunable *E_f_*, relaxation time (*τ*) is another key parameter of graphene; it reflects the average interval between carrier scattering events and directly determines carrier mobility and the complex conductivity of graphene. Although *τ* cannot be independently tuned purely by an electric field, it can be artificially tailored via multiple approaches, including controllable lattice defect engineering [10], temperature adjustment [11]. Combined with electrically tunable *E_f_*, cooperative regulation of the two parameters enables switching between narrowband and broadband absorption states on the same structural platform.

Driven by this potential of graphene for tunable THz absorption, researchers have devoted extensive efforts to the development of graphene-based tunable THz absorbers in recent years. Z. Li et al. designed a THz absorber with a gold–graphene–gold three-layer structure, achieving highly efficient absorption at five narrowband frequencies [12]. R. Zheng et al. proposed a THz absorber consisting of a gold–silicon dioxide–graphene architecture with triple-peak broadband absorption with an absorptivity exceeding 90% [13]. B. Zhang et al. developed a similar gold–silicon dioxide–graphene THz absorber, achieving narrowband absorption at six discrete frequencies [14]. Zhang et al. employed a patterned single-layer graphene metasurface combined with a polytetrafluoroethylene dielectric layer and a gold substrate, realizing a broadband absorption efficiency above 90% in the frequency range of 3.287–5.247 THz [15]. These studies confirm graphene’s potential for THz absorption, while they still face critical unresolved bottlenecks, i.e., the above-mentioned THz absorbers most rely on noble metals (e.g., Au, Ag) as the reflective layers, leading to high fabrication costs. Furthermore, few designs can simultaneously realize reversible narrowband–broadband absorption switching and maintain near-perfect absorption (absorptivity ≥ 99%).

To overcome the aforementioned bottlenecks (high cost and lack of switchable broadband and narrowband absorption), this work proposes a sandwich-structured THz absorber composed of a tungsten bottom reflective layer, a polyimide (PI) middle dielectric layer, and a patterned graphene top absorption layer. Compared with gold and silver, tungsten maintains satisfactory THz reflectivity while greatly reducing material costs and improving CMOS process compatibility, despite slightly higher intrinsic material loss. Meanwhile, compared with traditional metal-based and metamaterial absorbers, the proposed device realizes the dynamic tuning of THz absorption bandwidth via intrinsic parameters modulation of graphene. Moreover, it possesses comprehensive advantages including a simple structural design, compact volume, polarization insensitivity, and wide-angle absorption. Employing the finite element method (FEM), this work focuses on revealing the synergistic regulation mechanism of graphene’s *E_f_* and *τ* on the absorption performance within the 0–6 THz range. When *E_f_* and *τ* are 0.9 eV and 0.1 ps respectively, the proposed THz absorber exhibits two narrowband absorptions with a maximum absorptivity of 99.8%. In contrast, when *E_f_* and *τ* are 0.9 eV and 0.05 ps respectively, the proposed THz absorber achieves a 2.5 THz ultra-broad absorption bandwidth (defined by absorptivity ≥ 90%), including a 1.4 THz near-perfect absorption window. Meanwhile, the influences of key structural parameters, *E_f_*, *τ*, polarization angle, and incident angle on the absorption characteristics are systematically investigated. Mechanistically, the electromagnetic absorption mechanism is thoroughly elucidated by analyzing the electric field amplitude distributions of the THz absorber.

## 2. Theoretical Analysis

### 2.1. The Optical Conductivity Model of Graphene

As a two-dimensional Dirac semimetal, graphene’s surface conductivity consists of intraband ($\sigma_{\mathrm{intra}}(\omega )$) and interband ($\sigma_{\mathrm{inter}}(\omega )$) conductivities. According to the Kubo formula, the graphene’s conductivity (*σ*(*ω*)) can be expressed as the sum of these two components [16].

(1) $$\sigma (\omega )=\sigma_{\mathrm{intra}}(\omega )+\sigma_{\mathrm{inter}}(\omega )$$

At room temperature, when $\omega \ll k_{B}T/\hbar$ (ω=2πf denotes the angular frequency of THz waves, *f* is the incident wave frequency, $k_{B}$ refers to the Boltzmann constant, T means the absolute temperature, and ℏ is the reduced Planck constant), and $E_{f}\;\gg {\;k}_{B}T$; the contribution of interband transitions can be neglected. At this time, the optical conductivity of graphene is mainly dominated by intraband transitions, and its expression can be simplified as:

(2) $$\sigma_{\mathrm{intra}}(\omega )\;=\;\frac{e^{2}E_{f}\tau }{\pi \hbar^{2}(1\;+\;j\omega \tau )}$$

where e is the elementary charge (*e* = 1.602 × 10^−19^ C). It is evident that the intraband conductivity of graphene is in a complex form. The real part corresponds to the conductivity loss (directly related to the wave absorption performance), and the imaginary part corresponds to the admittance (affecting impedance matching).

Conversely, when $E_{f}\;<\;\hbar \omega /2$, the interband transitions cannot be ignored, and the expression for the optical conductivity is:

(3) σinter(ω)= e24ℏ[2ωω2+(2τ)2arctan(τ4Ef2 − (ℏω)22) + jln((ℏω)2+(2τ)24Ef2+(2τ)2)]

### 2.2. The Electromagnetic Response of Graphene Metamaterial Units

Based on the optical conductivity characteristics of graphene, the graphene metamaterial absorber designed in this paper adopts a sandwich structure of metal reflective layer-dielectric spacer layer–graphene metamaterial top layer. The top layer of the graphene metamaterial consists of periodic subwavelength units, whose core function is to enhance the interaction between THz waves and graphene through the resonance effect of the unit structure, thereby further improving the absorption performance and broadening the absorption bandwidth.

According to the transmission line theory, the equivalent impedance *Z_eff_* of the graphene metamaterial unit is jointly determined by the optical conductivity of graphene and the parameters of the unit structure. Considering that graphene is a two-dimensional thin layer and its thickness is much smaller than the THz wavelength, the influence of thickness can be ignored, so its equivalent surface impedance *Z_s_* is the reciprocal of the optical conductivity [17]:

(4) $$Z_{s}\;=\;\frac{1}{\sigma \left(\omega \right)}$$

The total equivalent impedance *Z_total_* of the absorber needs to match the free-space impedance *Z*_0_ = 377 Ω (*Z_total_* ≈ *Z*_0_). At this point, the reflection loss of the incident THz wave is the minimum and the absorption efficiency is the highest. The thickness *d* and dielectric constant *ε_r_* of the dielectric spacer layer can adjust the impedance matching state, and its equivalent transmission matrix *T* is:

(5) $$T\;=\;\left(\begin{array}{cc} \cos\left(\beta d\right) & jZ_{0}\sin\left(\beta d\right)/\sqrt{\varepsilon_{r}}\\ j\sqrt{\varepsilon_{r}}\sin\left(\beta d\right)/Z_{0} & \cos\left(\beta d\right) \end{array}\right)$$

where $\beta \;=\;\omega \sqrt{\mu_{0}\varepsilon_{0}\varepsilon_{r}}$ is the propagation constant of THz waves in the medium. $\mu_{0}$ and $\varepsilon_{0}$ represent the vacuum permeability and dielectric constant respectively. By adjusting the parameters of the dielectric spacer layers, the equivalent impedance of the absorber can be optimally matched.

### 2.3. Calculation of the Absorption Efficiency of the Absorber

The absorption efficiency $A\left(\omega \right)$ of the THz absorber is determined by the power relationship of the incident wave, reflected wave and transmitted wave. Since the proposed absorber contains a metal reflective layer, it can achieve total reflection of the transmitted wave (transmittance *T* = 0), thus the absorption efficiency can be simplified as [18]:

(6) $$A\left(\omega \right)\;=\;1-R\left(\omega \right)$$

where $R\left(\omega \right)$ represents the reflectivity, which is determined by the difference between the total equivalent impedance of the absorber and the impedance of free space. Its expression is:

(7) $$R\left(\omega \right)\;=\;{\left|\frac{Z_{\mathrm{total}}-Z_{0}}{Z_{\mathrm{total}}+Z_{0}}\right|}^{2\;}$$

Based on the graphene optical conductivity model mentioned earlier and the derived results of the equivalent impedance of the metamaterial unit, the absorption efficiency $A\left(\omega \right)$ can be expressed as a function of $E_{f}$, *τ*, and the device structure parameters (such as the thickness *d* of the dielectric, the dielectric constant *ε_r_*, and the period *a* of the metamaterial unit, etc.):

(8) $$A\left(E_{f},\tau ,\omega \right)\;=\;1-{\left|\frac{\frac{1}{\sigma (E_{f},\tau ,\omega )}+jZ_{0}\tan(\beta d)/\sqrt{\varepsilon_{r}}-Z_{0}}{\frac{1}{\sigma (E_{f},\tau ,\omega )}+jZ_{0}\tan(\beta d)/\sqrt{\varepsilon_{r}}+Z_{0}}\right|}^{2}$$

The above equation indicates that for fixed structural parameters, tuning $E_{f}$ and *τ* modulates graphene’s optical conductivity, which in turn adjusts the equivalent impedance and reflectivity of the absorber, enabling dynamic control of both absorption efficiency and bandwidth. The quantitative relationship between absorption performance and equivalent impedance further indicates that impedance matching is the core criterion for achieving high absorption efficiency, which is systematically analyzed in the following section.

### 2.4. Impedance Matching Theory

The above transmission line theory provides an analytical method for calculating the equivalent impedance *Z* of the absorber, while the *S* parameter retrieval method is introduced here for numerical validation and practical impedance matching analysis, which directly uses the scattering parameters obtained from numerical simulation. When an electromagnetic wave is incident, its transmission matrix is expressed as [19]:

(9) $$T\;=\;[\begin{array}{cc} T_{11} & T_{12}\\ T_{21} & T_{22} \end{array}]\;=\;[\begin{array}{cc} \cos(\mathrm{nkd}) & -\frac{z}{k}\sin(\mathrm{nkd})\\ \frac{k}{z}\sin(\mathrm{nkd}) & \cos(\mathrm{nkd}) \end{array}]$$

where *k* = 2π/*λ* is the propagation constant of the incident wave in a vacuum. The equivalent dielectric constant and magnetic permeability of metamaterials are respectively *ε* = *n*/*z* and *μ* = *nz*, *n* and *z* refer to the refractive index and wave impedance of metamaterials, respectively. Furthermore, the relationship between *S* parameter and the transmission matrix *T* is as follows:

(10) $$S_{21}\;={\;S}_{12}\;=\;\frac{2}{T_{11}+T_{22}+({\mathrm{ikT}}_{12}+\frac{T_{21}}{\mathrm{ik}})}\;=\;\frac{1}{\cos(\mathrm{nkd})-\sin(\mathrm{nkd})}$$

(11) $$S_{11}=S_{22\;}=\frac{T_{22}-T_{11}+({\mathrm{ikT}}_{12}-\frac{T_{21}}{\mathrm{ik}})}{T_{11}+T_{22}+({\mathrm{ikT}}_{12}+\frac{T_{21}}{\mathrm{ik}})}=\frac{i}{2}(\frac{1}{z}-Z)\sin(\mathrm{nkd})$$

From the above two equations, the expression for the normalized impedance can be derived:

(12) $$Z=\sqrt{\frac{{(1+S_{11}(\omega ))}^{2}-{S_{21}(\omega )}^{2}}{{(1-S_{11}(\omega ))}^{2}-{S_{21}(\omega )}^{2}}}$$

When $S_{11}(\omega )$ and $S_{21}(\omega )$ are both zero, the free-space impedance *Z*_0_ is equal to the equivalent impedance *Z* of the absorber, and $R\left(\omega \right)$ reaches the minimum value of 0, thereby obtaining a nearly perfect absorptivity $A\left(\omega \right)$. At this time, the equivalent impedance is perfectly matched.

## 3. Design

**Figure 1:**
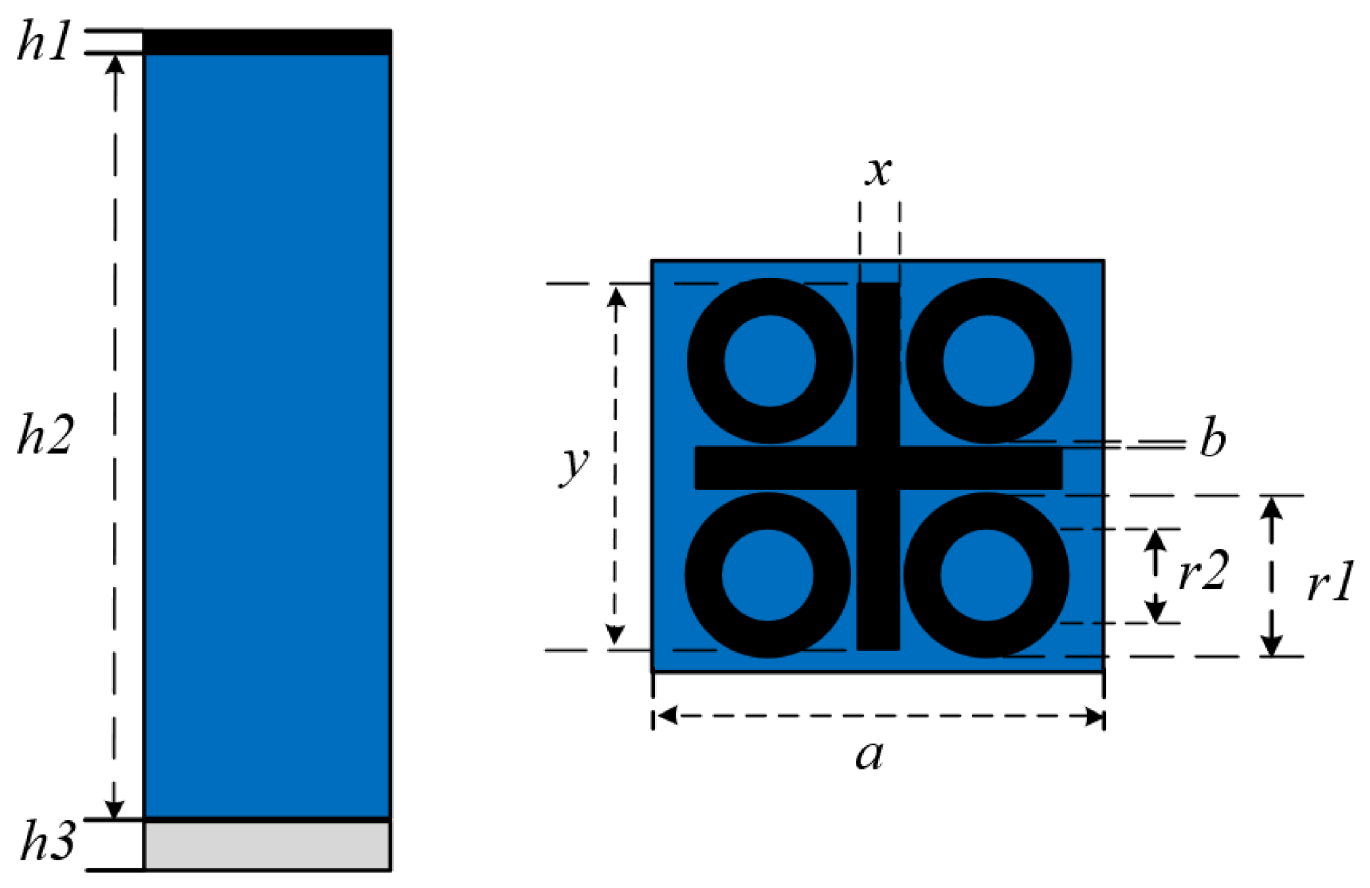
Architecture of the THz metamaterial absorber. (a) Front view. (b) Top view structural representation of the presented absorber.

To achieve broadband absorption and dynamic tunability in the THz regime, a graphene-based sandwich-structured metamaterial absorber is proposed. Note that, the numerical simulation and structural optimization of the presented THz absorber are implemented on the CST Microwave Studio 2023 platform. Periodic unit cell boundary conditions are assigned along the x- and y-axes to emulate an infinite planar metamaterial array. Along the wave propagation direction (*z*-axis), open boundaries are adopted to eliminate spurious reflected signals. A linearly polarized electromagnetic wave with x-direction electric field component is normally incident onto the absorber along the positive *z*-axis for full-wave calculation. As shown in Figure 1, the absorber has three layers: a top graphene metamaterial layer, a middle PI dielectric spacer, and a bottom tungsten reflective substrate. The proposed structure enables highly efficient THz absorption by combining impedance matching design of the dielectric layer, total reflection of the bottom substrate, and resonance enhancement of the top metamaterial. Specifically, the top graphene metamaterial acts as the core component for wave absorption and dynamic tuning, the middle PI layer is critical for realizing optimal impedance matching, and the bottom tungsten layer serves as a reflective substrate.

In numerical modeling at T = 300 K, the geometric thickness of graphene is set to *h*1 = 1 nm. This value is an equivalent thickness only for mesh partitioning in simulation. Considering the weak interaction between pristine single-layer graphene and THz waves, the graphene is patterned into a periodic subwavelength structure composed of a cross and four concentric rings and the structure exhibits a high degree of geometric symmetry, providing a structural basis for the polarization-insensitive characteristic of the device. The specific design of the composite metamaterial unit is as follows: A cross structure is positioned at the center. Considering the characteristics of the THz frequency regime and optimizing the dimensions based on the 22 μm unit cell period (*a*), the cross arms are designed with a width of 3 μm (*x*) and a length of 18 μm (*y*). Four identical concentric rings are symmetrically distributed around the cross. Each concentric ring has an inner diameter of 2 μm (*r*2), an outer diameter of 4 μm (*r*1), and a line width of 2 μm, which matches the width of the cross arms and is compatible with the 22 μm unit cell period. The spacing between adjacent rings and the cross is uniform (*b* = 0.1 μm).

The patterned graphene metamaterial serves as the core of the absorber, while the middle dielectric spacer layer is critical for optimizing impedance matching, which is made of PI (the permittivity of *ε_r_* is 3.5) with an optimized thickness of 11 μm (*h*2). Its core function is to regulate the equivalent impedance Z of the absorber, achieve good matching with the free-space impedance (*Z*_0_ = 377 Ω), reduce the reflection loss of THz waves, and improve the absorption efficiency. PI exhibits excellent thermal stability, mechanical flexibility, and low THz loss characteristics, with a stable relative permittivity [20].

Complementing the top and middle layers, the bottom reflective layer ensures total reflection of THz waves, completing the sandwich structure design. The bottom layer employs metallic tungsten (the conductivity of *σ* is 1.89 × 10^7^ S/m) as the reflective layer with a designed thickness of 0.2 μm (*h*3). It is noted that, compared with conventional Au and Ag reflective layers widely adopted in reported THz absorbers, tungsten exhibits competitive THz reflectivity, lower material cost and better compatibility with CMOS fabrication processes [21]. Although tungsten introduces slightly higher intrinsic THz loss than noble metals, the designed thickness of 0.2 μm exceeds its THz skin depth sufficiently, ensuring near-complete reflection without degrading absorption performance. This ensures that the absorption efficiency satisfies the simplified relation $A\left(\omega \right)\;=1-\;R\left(\omega \right)$ with transmittance *T* = 0 and maximizes the absorption efficiency.

## 4. Results and Discussion

**Figure 2:**
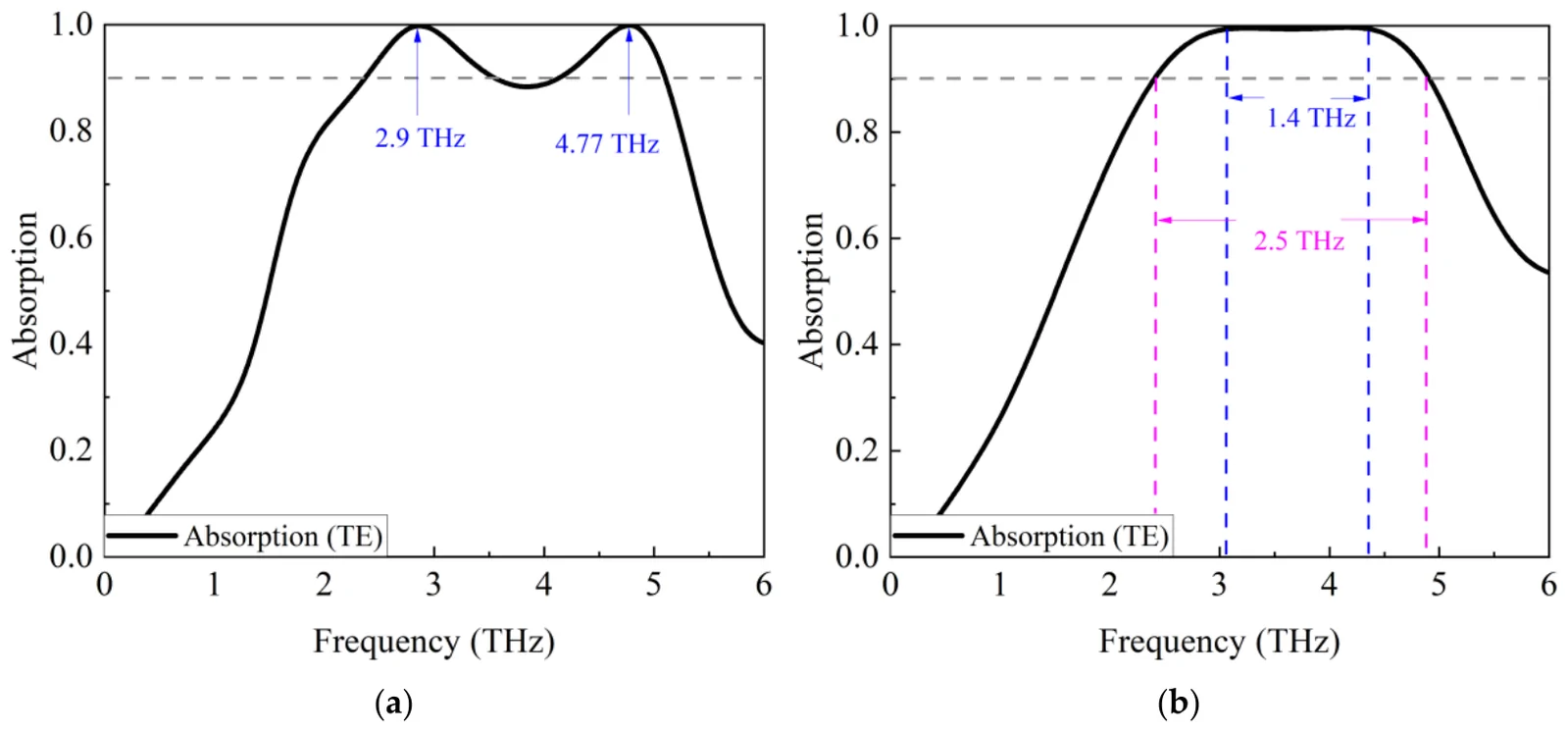
The obtained absorption spectra in the case of $E_{f}$ = 0.9 eV and (**a**) *τ* = 0.1 ps; (**b**) *τ* = 0.05 ps.

Figure 2a shows the absorption spectra of the proposed absorber in the THz regime (0–6 THz) when $E_{f}$ is 0.9 eV and *τ* is 0.1 ps. Endowed with rotational symmetry, the proposed structure exhibits identical absorption spectra for both TE and TM polarizations under normal electromagnetic wave incidence. Consequently, TE polarization is selected as the representative case for subsequent investigations. As can be observed, the absorber exhibits a narrowband absorption response under this condition. In particular, at two characteristic frequencies of 2.9 THz and 4.77 THz, the absorptivity approaches nearly 100%, indicating that the absorber achieves highly efficient narrowband absorption at specific frequencies. This phenomenon can be attributed to a relatively long relaxation time (*τ* = 0.1 ps), which reduces the carrier scattering probability and improves the carrier mobility in graphene, thereby increasing the sharpness of the frequency response of its optical conductivity. Consequently, the resonance effect of the graphene metamaterial unit (the cross-four-ring composite structure) becomes more concentrated, enabling efficient absorption and energy dissipation of incident THz waves only at discrete resonant frequencies. This ultimately leads to a narrowband, high-efficiency absorption performance.

When *τ* is reduced to 0.05 ps while the $E_{f}$ remains unchanged at 0.9 eV, the absorption characteristics of the absorber undergo a significant transition, as illustrated in Figure 2b. The device switches from narrowband absorption to broadband absorption. It can be seen that the absorptivity remains close to 100% within a wide frequency range of 1.4 THz. Meanwhile, the absorber achieves high-efficiency THz absorption (≥90%) over a bandwidth of 2.5 THz. The key to this performance transition lies in the regulation of *τ*. Reducing *τ* enhances carrier scattering in graphene and shortens the carrier lifetime, which significantly broadens the frequency response bandwidth of the optical conductivity so that it is no longer restricted to one or a few discrete resonant frequencies. Meanwhile, the widely tunable optical conductivity cooperates with the superposition effect of the high- and low-frequency resonances introduced by the composite metamaterial structure. This synergistic effect allows the absorber to maintain excellent impedance matching with free space over a broad frequency range, thus reducing reflection loss. The energy of incident THz waves is then efficiently converted into thermal energy through the carrier relaxation process, ultimately achieving broadband, high-efficiency THz absorption.

**Figure 3:**
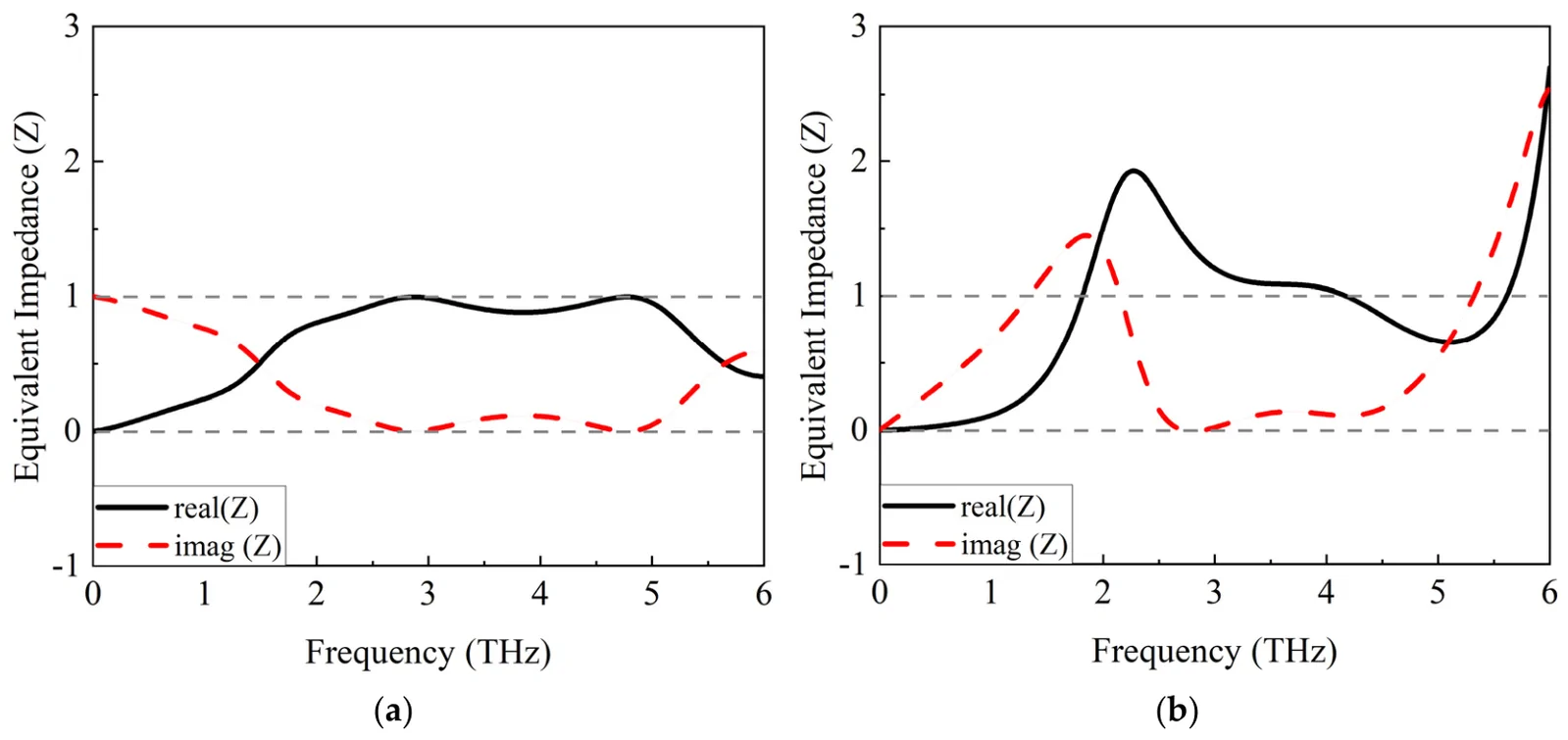
Equivalent impedance *Z*’s real and imaginary parts in the case of $E_{f}$ = 0.9 eV and (**a**) *τ* = 0.1 ps; (**b**) *τ* = 0.05 ps.

Based on the analysis in the theoretical part of 2.4, we can also explain the absorption mechanism of this absorber using the impedance matching principle. As shown in Figure 3a, at *τ* = 0.1 ps, the real part of the equivalent impedance *Z* approaches 1 and the imaginary part approaches 0 exactly at the two resonant peaks (2.9 THz and 4.77 THz), confirming that perfect impedance-matching dominates the narrowband high-efficiency absorption. For *τ* = 0.05 ps (Figure 3b), excellent impedance matching is maintained across the entire 2.4 to 4.9 THz band, which is highly consistent with the measured 2.5 THz broadband absorption.

**Figure 4:**
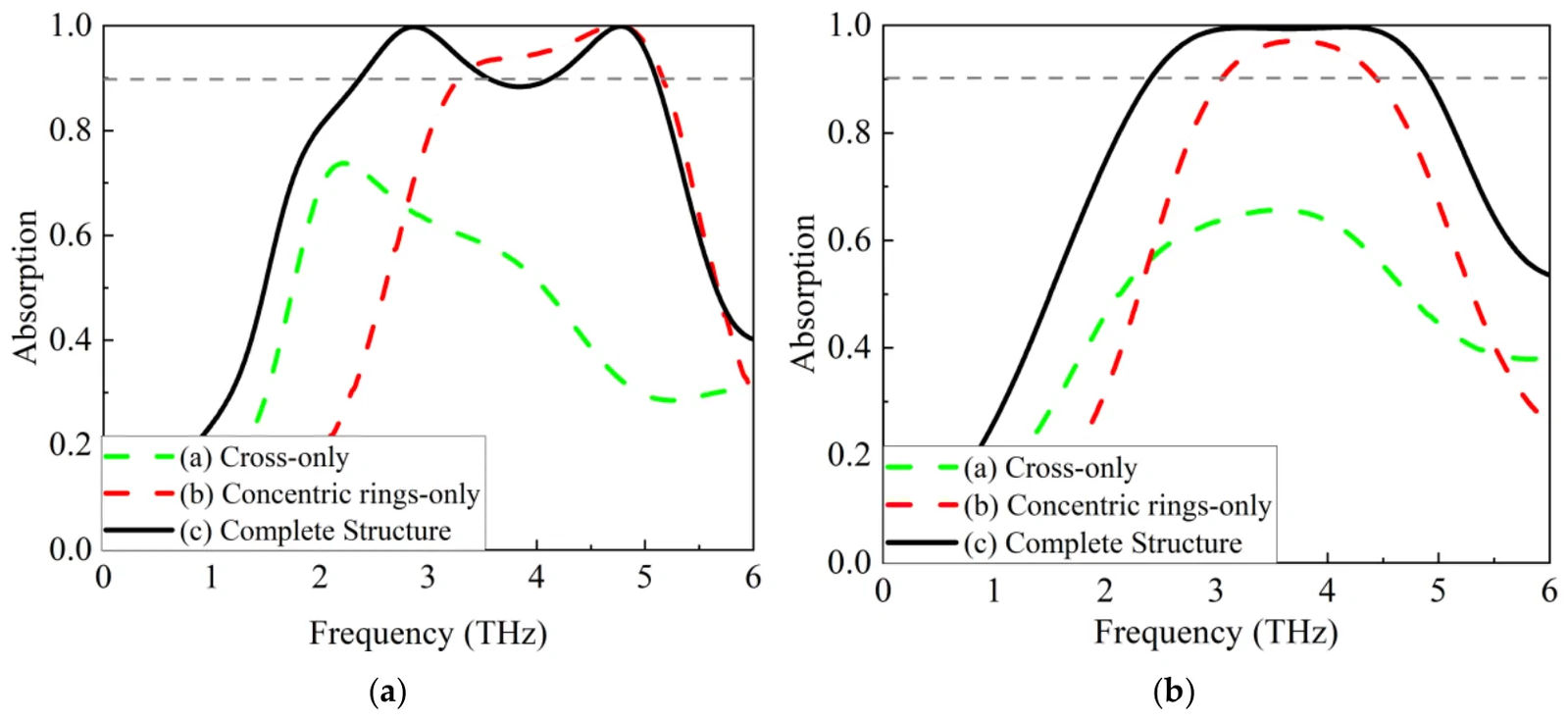
Absorption curves with cross-only, concentric rings-only or complete structure in the case of (**a**) $E_{f}$ = 0.9 eV and *τ* = 0.1 ps; (**b**) $E_{f}$ = 0.9 eV and *τ* = 0.05 ps.

In order to study the absorption performance of the absorber under different graphene patch structures (cross-only, concentric rings-only, and complete structure), we calculated three scenarios under normal incidence of electromagnetic waves. From Figure 4a, it can be seen that when $E_{f}$ = 0.9 eV and *τ* = 0.1 ps and the graphene patch is cross-only structure, it shows that only a single absorption peak at a low frequency, with a narrow absorption bandwidth and a peak absorptivity around 70%. This indicates that the resonance effect of the single cross pattern is relatively weak, enabling only limited absorption within a specific low-frequency range. The concentric rings-only structure exhibits a certain degree of narrowband absorption, but it has fewer absorption peaks and a lower absorptivity compared to the complete structure, suggesting that although the single ring structure can excite high-frequency resonance, it lacks synergistic interaction with low-frequency resonance. In sharp contrast, the complete structure displays typical dual-band and high-efficiency absorption performance. Especially, the absorptivity approaches nearly 100% at two characteristic frequencies of 2.9 THz and 4.77 THz. We can infer that the combination of the two (structural coupling effect) realizes the dual-band and high-efficiency absorption performance. Meanwhile, benefiting from the sharp frequency response of graphene’s optical conductivity at *τ* = 0.1 ps, the absorption efficiency at specific frequencies is further enhanced, ultimately achieving dual-band high-efficiency absorption.

Figure 4b depicts the THz absorption spectra of the absorber with different structural configurations at $E_{f}$ = 0.9 eV and *τ* = 0.05 ps, where the structural types are consistent with those in Figure 4a. Similar to the performance of *τ* = 0.1 ps case, the cross-only structure exhibits a narrow absorption bandwidth and a peak absorptivity below 70%. The concentric rings-only structure also possesses a certain degree of narrowband absorption, but it has a lower absorptivity compared to the complete structure. In contrast, the complete structure demonstrates favorable broadband and high-efficiency absorption performance. It can be seen that, within the 2.5 THz frequency range, the absorption efficiency of the absorber is always greater than 90%. Particularly, within a continuous frequency range of 1.4 THz, the absorptivity remains consistently close to 100%, and the absorption curve is smooth without noticeable attenuation, which is far superior to the performance of the two individual structures. The core reason for this distinction lies in the changed optical response of graphene at *τ* = 0.05 ps. The enhanced carrier scattering in graphene significantly broadens the frequency response range of its optical conductivity, which provides favorable conditions for the superposition of low- and high-frequency resonances in the complete structure. The synergistic effect between the cross and the four concentric rings enables the resonant bands of the two components to connect and overlap with each other, eliminating the absorption gaps inherent in individual structures, thus achieving high-efficiency absorption over a broad frequency range. In contrast, due to the lack of such synergistic interaction, the individual structures cannot simultaneously improve both the absorption bandwidth and absorption efficiency.

**Figure 5:**
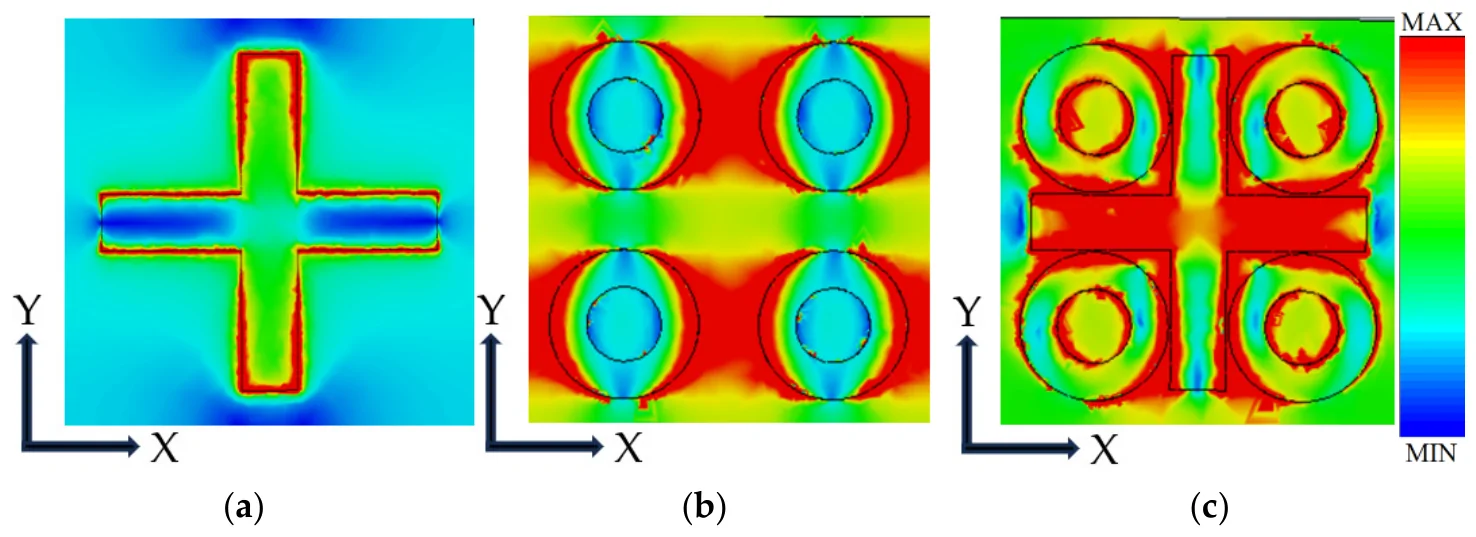
Normalized electric field distribution at 2.9 THz for the absorber with (**a**) cross-only (**b**) concentric rings-only (**c**) complete structure when the $E_{f}$ = 0.9 eV and *τ* = 0.1 ps.

**Figure 6:**
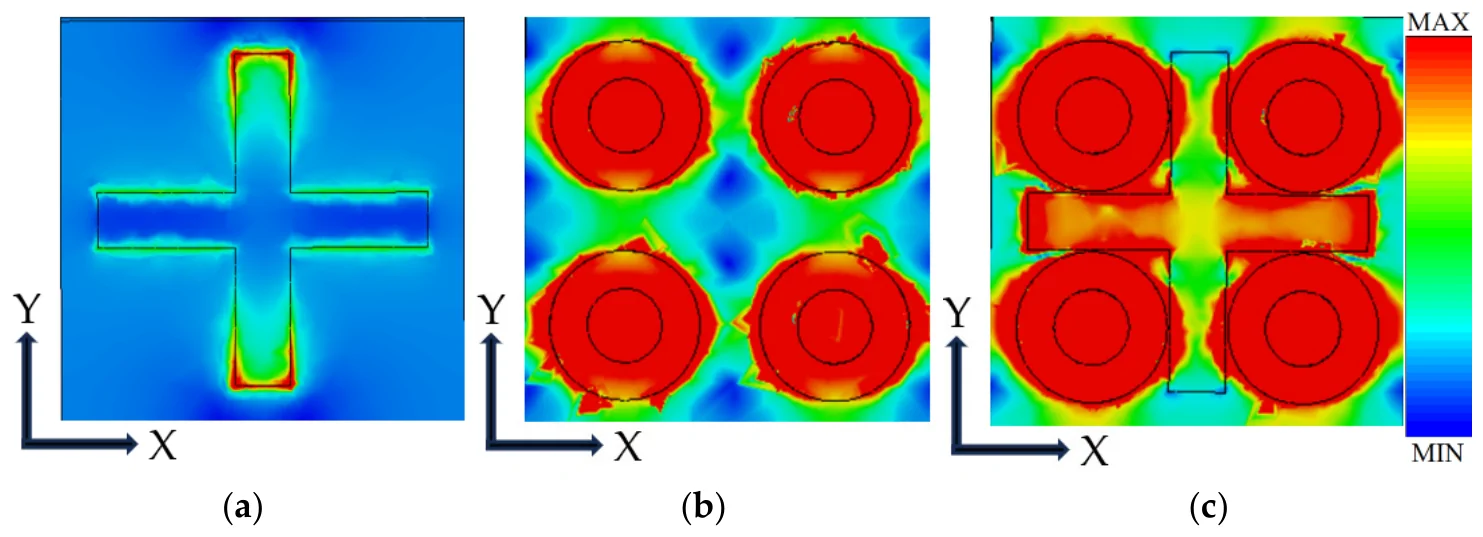
Normalized electric field distribution at 4.2 THz for the absorber with (**a**) cross-only (**b**) concentric rings-only (**c**) complete structure when the $E_{f}$ = 0.9 eV and *τ* = 0.05 ps.

Figure 5a–c display the normalized electric field distributions of the absorber with different configurations under the narrowband absorption mode ($E_{f}$ = 0.9 eV and τ *=* 0.1 ps), corresponding to the cross-only structure (Figure 5a), concentric rings-only structure (Figure 5b), and the complete structure (Figure 5c), respectively. Note that, the MIN = 0 (zero electric field amplitude, unit: V/m) and MAX = 1 (normalized peak electric field amplitude, corresponding to the maximum raw |E| magnitude in V/m within the unit cell at the target frequency) in Figure 5 and Figure 6. Due to that, the absorption efficiency at 2.9 THz and 4.77 THz is almost identical; the normalized electric field distributions of 2.9 THz are discussed as an example. As shown in Figure 5a, the normalized electric field is mainly concentrated at the edges and endpoints of the cross arms, with an inhomogeneous distribution and a narrow strong-field region. Obvious energy dissipation only occurs in local areas of the cross structure. Combined with the aforementioned absorption spectra, this structure only provides weak absorption, with a peak absorptivity around 70%. This behavior is directly related to the normalized electric field distribution, i.e., the narrow and inhomogeneous strong-field region results in limited energy dissipation, which cannot support efficient absorption of incident THz waves, consistent with the weak resonance effect of the cross-only structure. Figure 5b shows a different normalized electric field distribution. The field is mainly concentrated at the outer edges and junctions of the concentric rings, forming a ring-shaped strong-field region. However, the normalized electric field intensity is lower than that of the complete structure. The underlying reason is that the strong-field region of the single ring structure is confined locally, and energy dissipation is concentrated only in the high-frequency resonant band. Without synergistic interaction with low-frequency resonance, the total energy dissipation is insufficient to achieve high-efficiency absorption. In sharp contrast, Figure 5c presents a dual-region strong normalized electric field distribution. Strong fields are concentrated at the cross arms and the outer and inner edges of the four rings simultaneously. The strong-field region is significantly expanded and more uniformly distributed, and the field intensity is markedly higher than those of the two individual structures, indicating that the complete structure realizes the synergistic superposition electric fields. Combined with the absorption spectra, the complete structure achieves near-100% absorptivity at 2.9 THz.

Here, the normalized electric field distributions of 4.2 THz are given as an example. Figure 6a–c plot the normalized electric field distributions of the absorber with various configurations under the broadband absorption mode ($E_{f}$ = 0.9 eV, *τ* = 0.05 ps). Similar to the above-mentioned results, the complete structure displays the optimal normalized electric field distribution and energy dissipation characteristics. The strong electric field area is completely expanded and seamlessly connected, covering most of the cross structures and the entire four-ring structures. The electric field is uniformly distributed with the highest overall intensity, and the energy dissipation remains strong and sustained over a wide frequency range. Combined with the absorption spectra, the complete structure achieves a high and stable absorptivity (≥90%) within the frequency band of 2.4–4.9 THz (bandwidth 2.5 THz). In particular, the absorption maintains almost 100% within a 1.4 THz continuous frequency window, realizing superior broadband and high-efficiency THz absorption.

**Figure 7:**
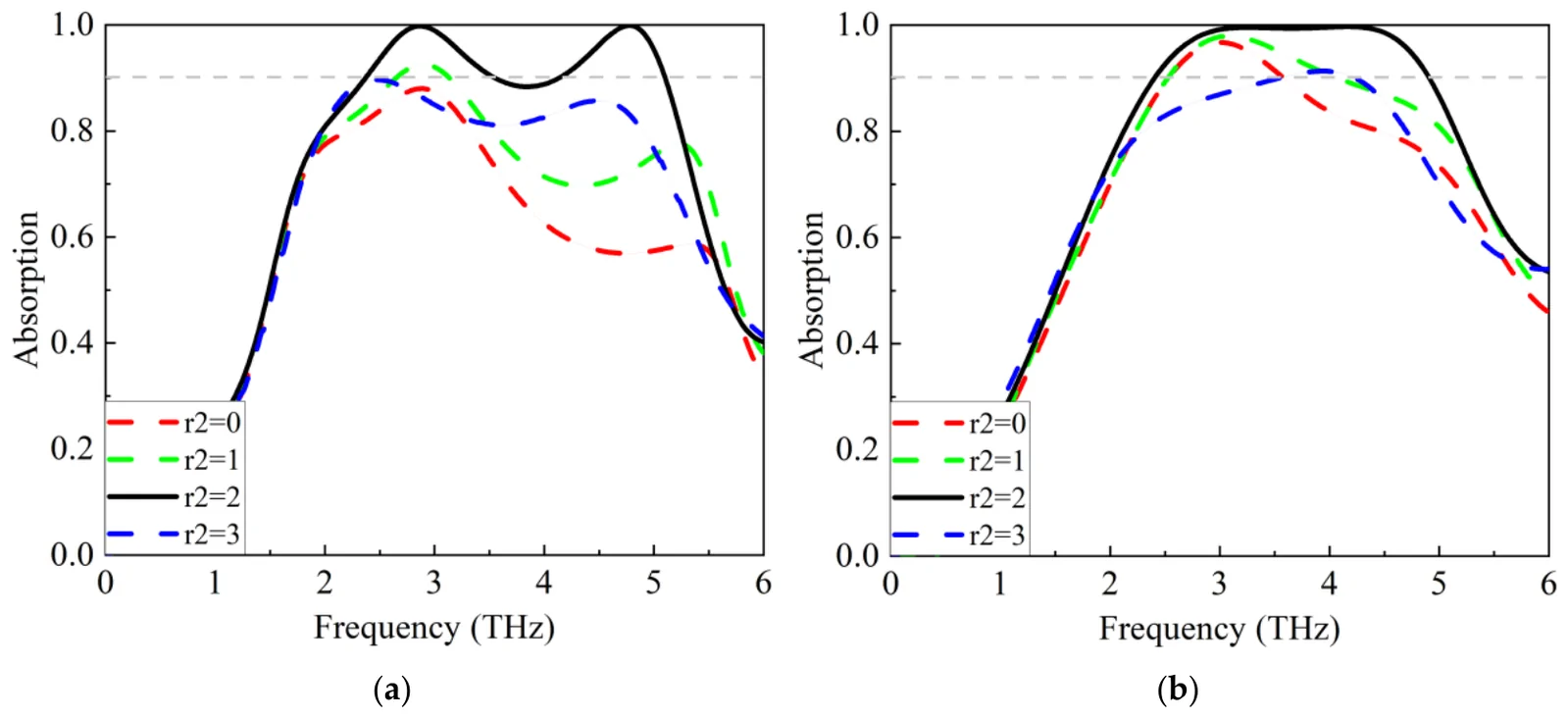
Absorption curves under different *r*2 values in the case of (**a**) $E_{f}$ = 0.9 eV and *τ* = 0.1 ps; (**b**) $E_{f}$ = 0.9 eV and *τ* = 0.05 ps.

In a defined THz frequency range, the impacts of diverse structural parameters on absorption behaviors will be studied independently. Figure 7a illustrates the absorption spectra of the THz absorber with different inner diameters (*r*2) at $E_{f}$ = 0.9 eV and *τ* = 0.1 ps. It can be clearly observed from the curves that the absorption performance of the absorber exhibits a strong dependence on the inner diameter. Specifically, as *r*2 increases, the resonant absorption peak gradually evolves from a single peak into two distinct peaks, accompanied by an obvious broadening of the resonant peaks. The maximum absorptivity is enhanced to nearly 100%, indicating optimized matching between the structural parameters and the incident THz waves. This behavior could be attributed to the fact that a larger *r*2 strengthens the spatial overlap between the incident electromagnetic field and the surface plasmon polariton (SPP) modes, which further promotes energy dissipation of electromagnetic waves inside the absorber and thus improves the absorption intensity. Figure 7b displays the absorption spectra under the same Fermi level ($E_{f}$ = 0.9 eV) but a shorter carrier relaxation time (*τ* = 0.05 ps). By comparing Figure 7b with Figure 7a, it is revealed that *τ* exerts a remarkable modulation effect on the absorption performance, while the dependence of the absorption spectra on the ring diameter follows the same trend as that observed in Figure 7a (i.e., the absorption intensity varies with increasing ring diameter).

**Figure 8:**
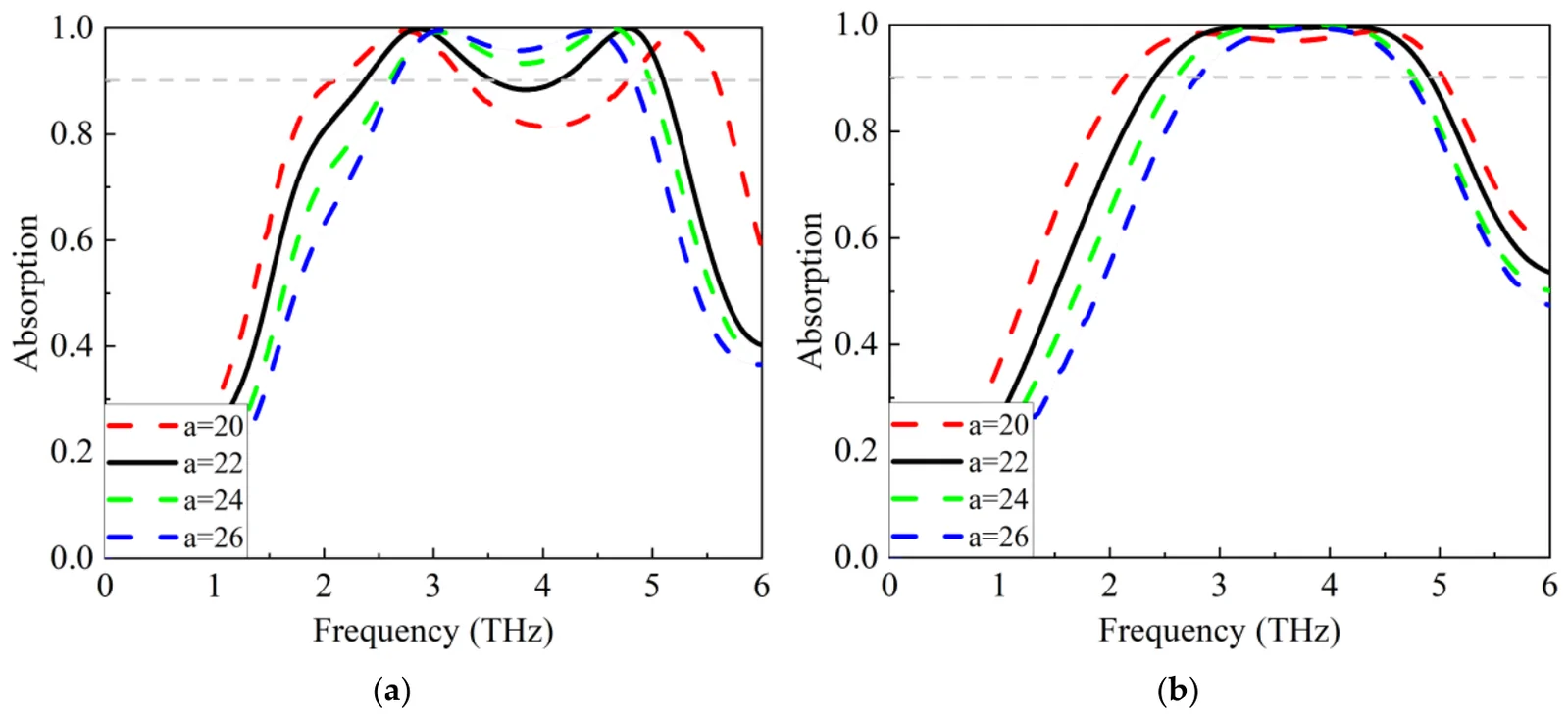
Absorption curves with different *a* values in the case of (**a**) $E_{f}$ = 0.9 eV and *τ* = 0.1 ps; (**b**) $E_{f}$ = 0.9 eV and *τ* = 0.05 ps.

The influence of the unit cell period *a* on the absorption spectrum is further studied. Figure 8a presents the absorption spectra of the THz absorber with different unit cell periods (*a* = 20, 22, 24, 26 μm) at $E_{f}$ = 0.9 eV and *τ* = 0.1 ps. It can be clearly observed from the curves that the absorber’s absorption performance exhibits a strong dependence on the unit cell period *a*. Moreover, varying the unit cell period provides a regular modulation effect on the position of the resonant absorption peaks. Specifically, as *a* increases from 20 μm to 26 μm, the low-frequency resonant absorption peaks undergo a blue-shift toward the high-frequency region, while the high-frequency resonant absorption peaks undergo an obvious red-shift toward the low-frequency region, keeping the absorption intensity almost relatively stable. It can be explained that the surface plasmon resonances (SPRs) generated at the interface can convert the incident energy into confined SPPs, which are then trapped or dissipated in the structure. Therefore, both the electric and Fabry–Perot resonances of the localized surface plasmon resonance (LSPR) are dominating the absorption, and the change in the geometrical parameters affects the above-mentioned processes, which makes the resonant frequency change [13]. Figure 8b shows the absorption spectra under the same Fermi level ($E_{f}$ = 0.9 eV) but a shorter carrier relaxation time (*τ* = 0.05 ps). Consistent with the *r*2-dependent trend, *τ* modulation remains effective for different *a* values, and the optimal absorption performance is achieved at *a* = 22 μm.

**Figure 9:**
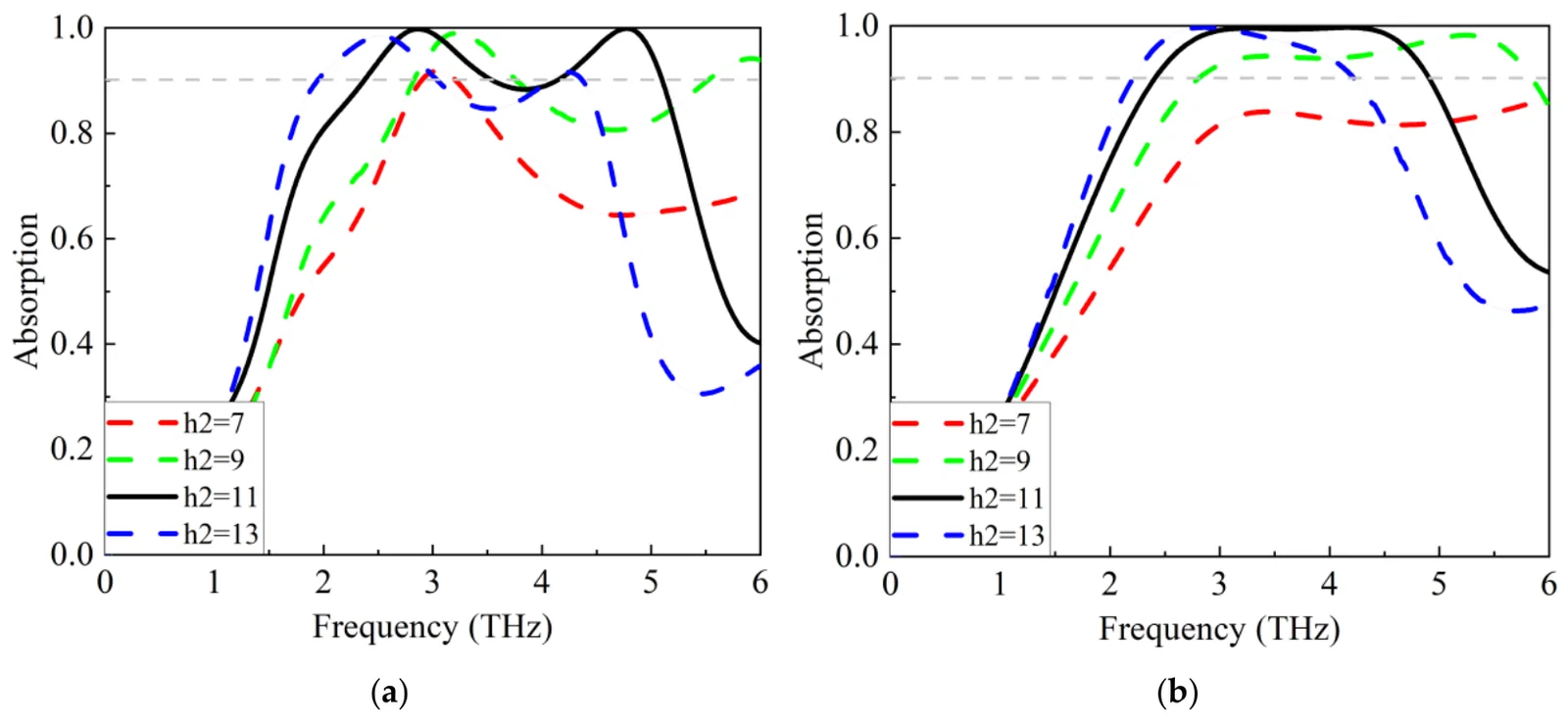
Absorption curves with different *h*2 values in the case of (**a**) $E_{f}$ = 0.9 eV and *τ* = 0.1 ps; (**b**) $E_{f}$ = 0.9 eV and *τ* = 0.05 ps.

The last parameter is the thickness of PI layer (*h*2). Figure 9a presents the absorption spectra of the THz absorber with different *h*2 values at $E_{f}$ = 0.9 eV and *τ* = 0.1 ps. It can be clearly observed from the curve distribution that as *h*2 increases, the resonant absorption peak of the absorber evolves from one to two, and the absorption intensity of the resonant peaks increase and then decrease. Furthermore, the frequency of the left resonant peak first undergoes a blue-shift and then a red-shift, while the frequency of the right resonant peak gradually shifts to the red. Figure 9b displays the absorption spectra of the absorber with different PI thicknesses under the same $E_{f}$ (0.9 eV) but a shorter carrier relaxation time (*τ* = 0.05 ps). The absorptivity first increases and then decreases with rising *h*2, reaching the optimum at *h*2 = 11 μm. Overall, the optimized structural parameters are determined as *r*2 = 2 μm, *a* = 22 μm, and *h*2 = 11 μm, which provide a design guideline for subsequent experimental fabrication.

**Figure 10:**
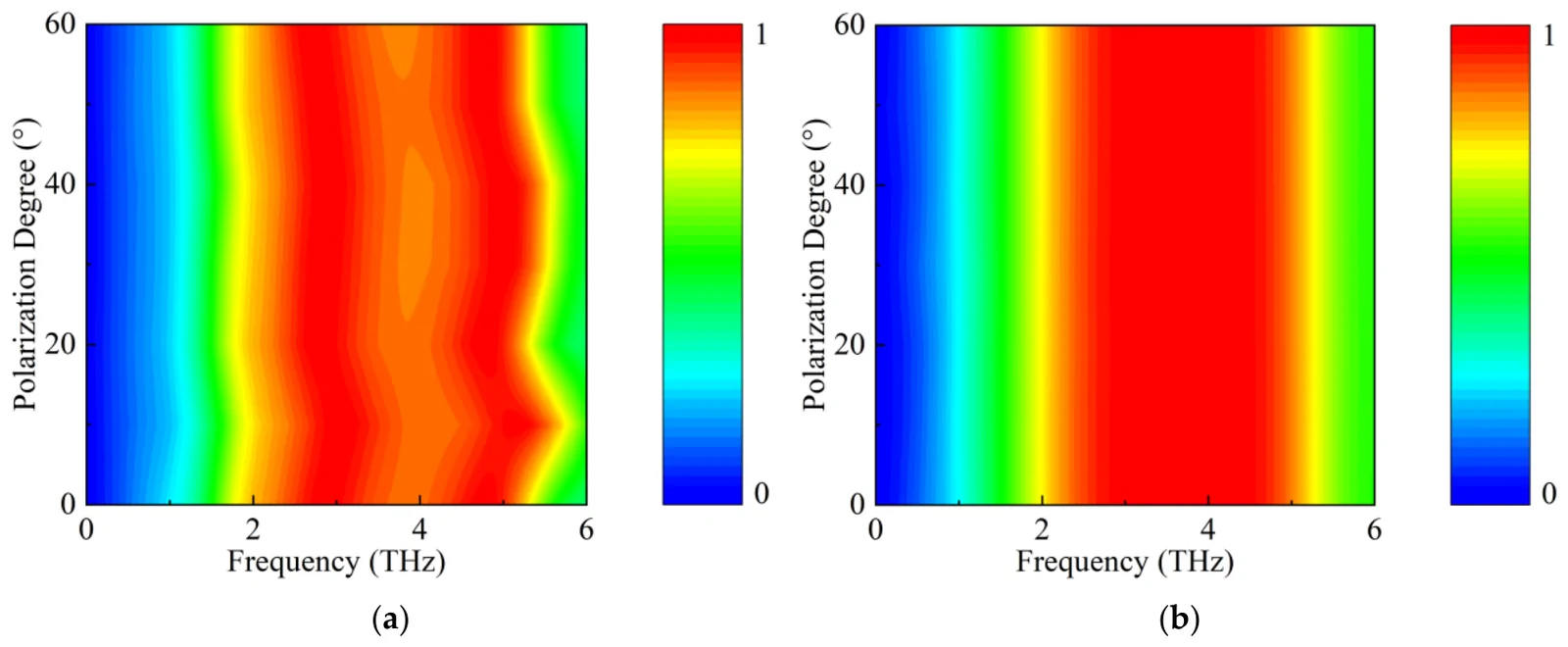
Simulated absorptivity plotted against frequency (*x*-axis) and polarization angle (*y*-axis) under the conditions of (**a**) $E_{f}$ = 0.9 eV and *τ* = 0.1 ps; (**b**) $E_{f}$ = 0.9 eV and *τ* = 0.05 ps.

For practical applications, the stability of absorption performance under varying polarization states and oblique incident angles is a critical indicator. For the proposed THz absorber, consistent absorption spectra are achieved for both TE and TM polarizations with the incident polarization angle changing from 0° to 60°. Thus, only the absorption spectra under TE polarization are provided herein. It can be seen from Figure 10a,b that there is no change in the absorption bandwidth or the corresponding absorptivity at various frequencies with the adjustment of the incident polarization angle under the condition of both $E_{f}$ = 0.9 eV, *τ* = 0.1 ps and $E_{f}$ = 0.9 eV and *τ* = 0.05 ps. The perfect polarization insensitivity of the absorber originates from its high geometric symmetry.

**Figure 11:**
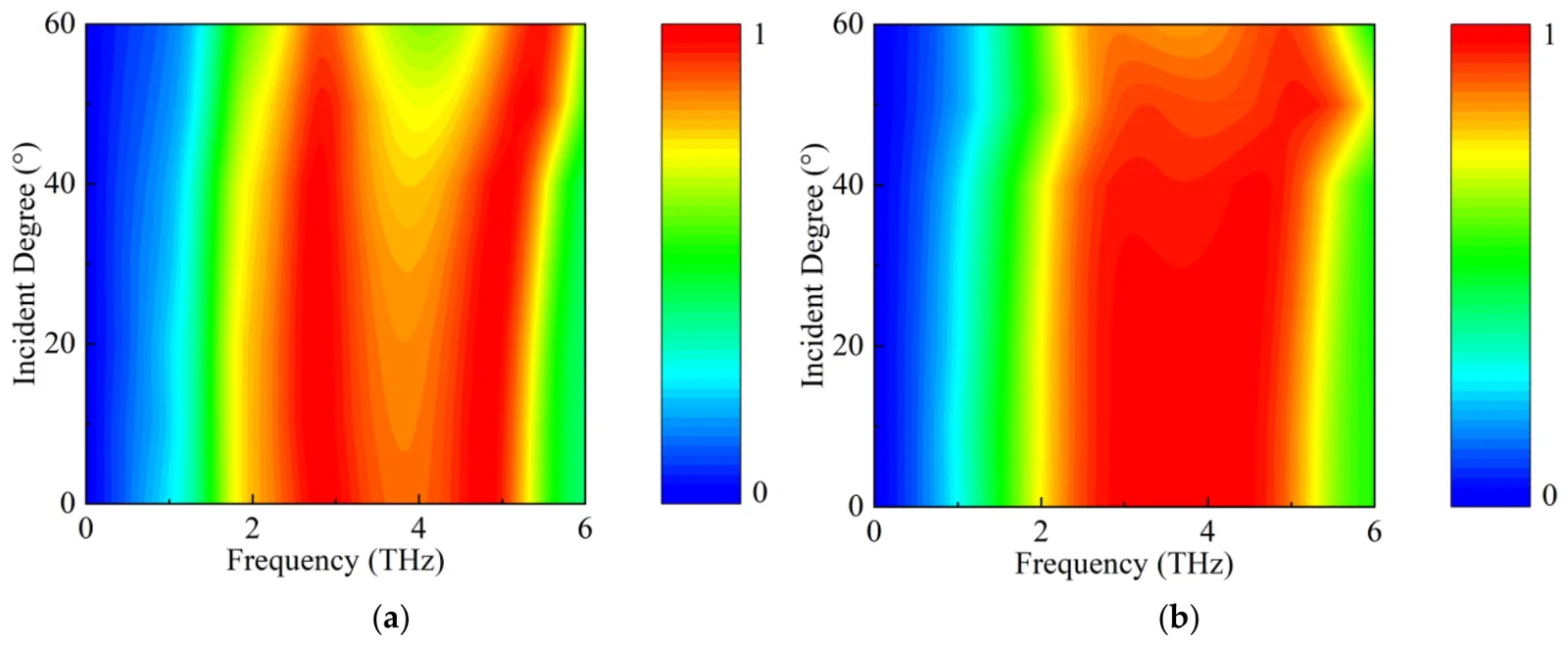
Simulated absorptivity plotted against frequency (*x*-axis) and incident angle (*y*-axis) at the case of (**a**) $E_{f}$ = 0.9 eV and *τ* = 0.1 ps; (**b**) $E_{f}$ = 0.9 eV and *τ* = 0.05 ps.

Further research is carried out to assess how oblique incidence angles influence the absorptivity. Figure 11a shows the absorption spectra of the THz absorber for various incident angles at $E_{f}$ = 0.9 eV, *τ* = 0.1 ps. It can be seen that, at small incident angles (0°, normal incidence), the absorber achieves the optimal absorption performance in the resonant band, with the maximum absorptivity approaching 100% (near-perfect absorption), while maintaining high absorption over a relatively wide frequency range. Then, although with the incident angle increases gradually from 0° to 60°, the absorption intensity of the absorber exhibits a gradual decreasing trend, the absorptivity of the dual-band remains above 90%. Figure 11b presents the absorption spectra under the same $E_{f}$ (0.9 eV) but a shorter carrier relaxation time (*τ* = 0.05 ps) for different incident angles. It is found that the dependence of the absorption spectra on the incident angle is generally consistent with the variation trend observed in Figure 11a. Collectively, the designed absorber possesses wide-angle absorption properties, thereby presenting considerable value for real-world applications.

**Figure 12:**
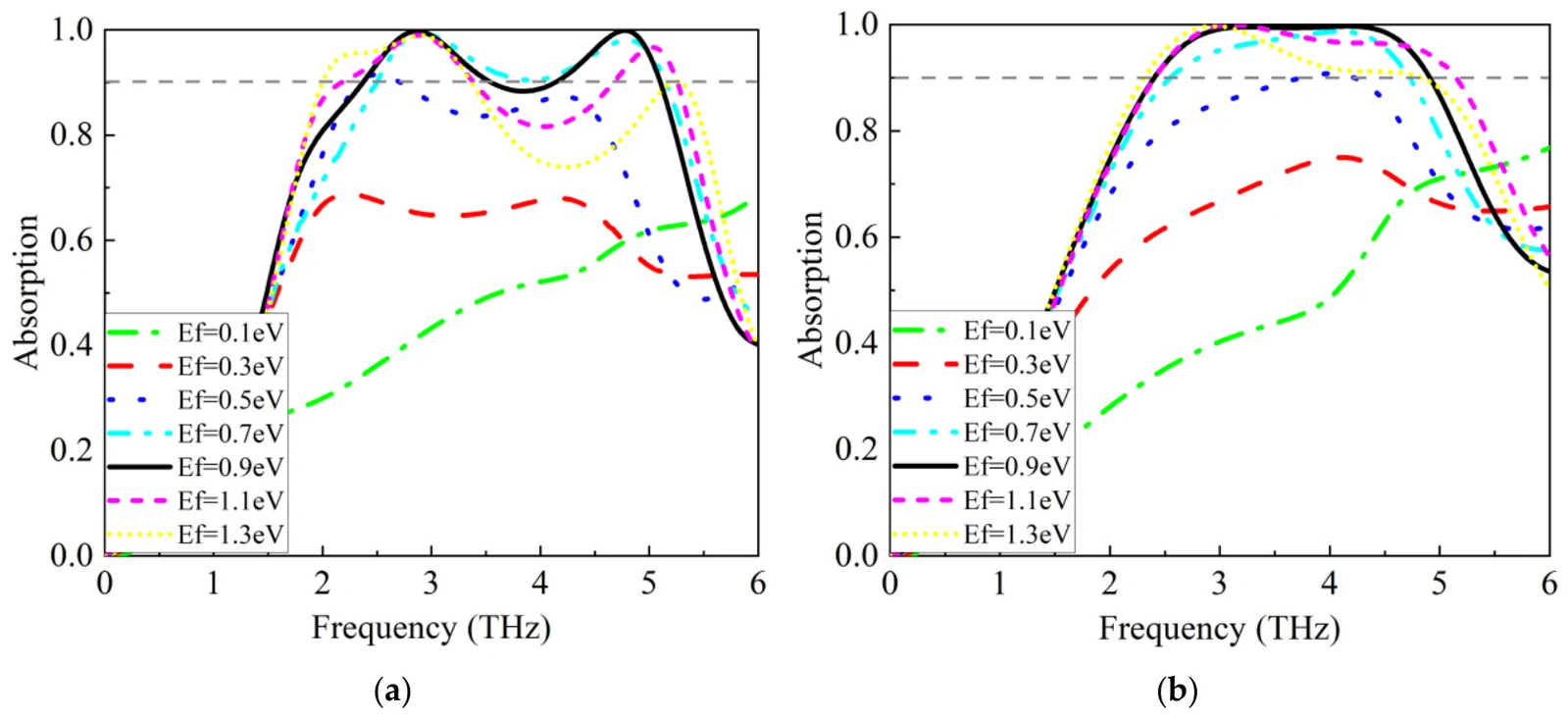
Absorption curves with different $E_{f}$ values in the case of (**a**) *τ* = 0.1 ps; (**b**) *τ* = 0.05 ps.

Finally, the tunable properties of graphene are investigated. As mentioned earlier, as a widely tunable material, graphene plays a crucial role in studies related to THz. Figure 12a presents the absorption spectra of the THz absorber for different $E_{f}$ (0.1 eV, 0.3 eV, 0.5 eV, 0.7 eV, 0.9 eV, 1.1 eV and 1.3 eV) at *τ* = 0.1 ps. It can be seen that the absorption performance of the absorber exhibits a strong dependence on $E_{f}$. Moreover, the continuous variation of $E_{f}$ provides a regular modulation effect on the position, intensity, and bandwidth of the resonant absorption peaks. Specifically, as $E_{f}$ increases from 0.1 eV to 1.3 eV, the absorption intensity of the absorber first increases significantly and then decreases. At a low $E_{f}$ (0.1 eV), the absorber shows extremely weak absorption across the entire simulated frequency band, with the maximum absorptivity below 70% and no distinct resonant absorption peaks. As $E_{f}$ gradually rises from 0.1 eV to 0.9 eV, the resonant absorption peaks emerge and become increasingly pronounced, and the maximum absorptivity rapidly increases to nearly 100%. This indicates that the increase in $E_{f}$ significantly improves the energy absorption and conversion efficiency of the absorber. When $E_{f}$ is further increased to 1.1 eV and 1.3 eV, the absorption intensity shows no obvious improvement and tends to decline. Figure 12b displays the absorption spectra of the absorber for different $E_{f}$ under a shorter carrier relaxation time (*τ* = 0.05 ps). By comparing Figure 12b with Figure 12a, it is revealed that the relaxation time *τ* exerts a remarkable modulation effect on the absorption performance, while the dependence of the absorption spectra on the Fermi level follows the same trend as that observed in Figure 12a.

**Figure 13:**
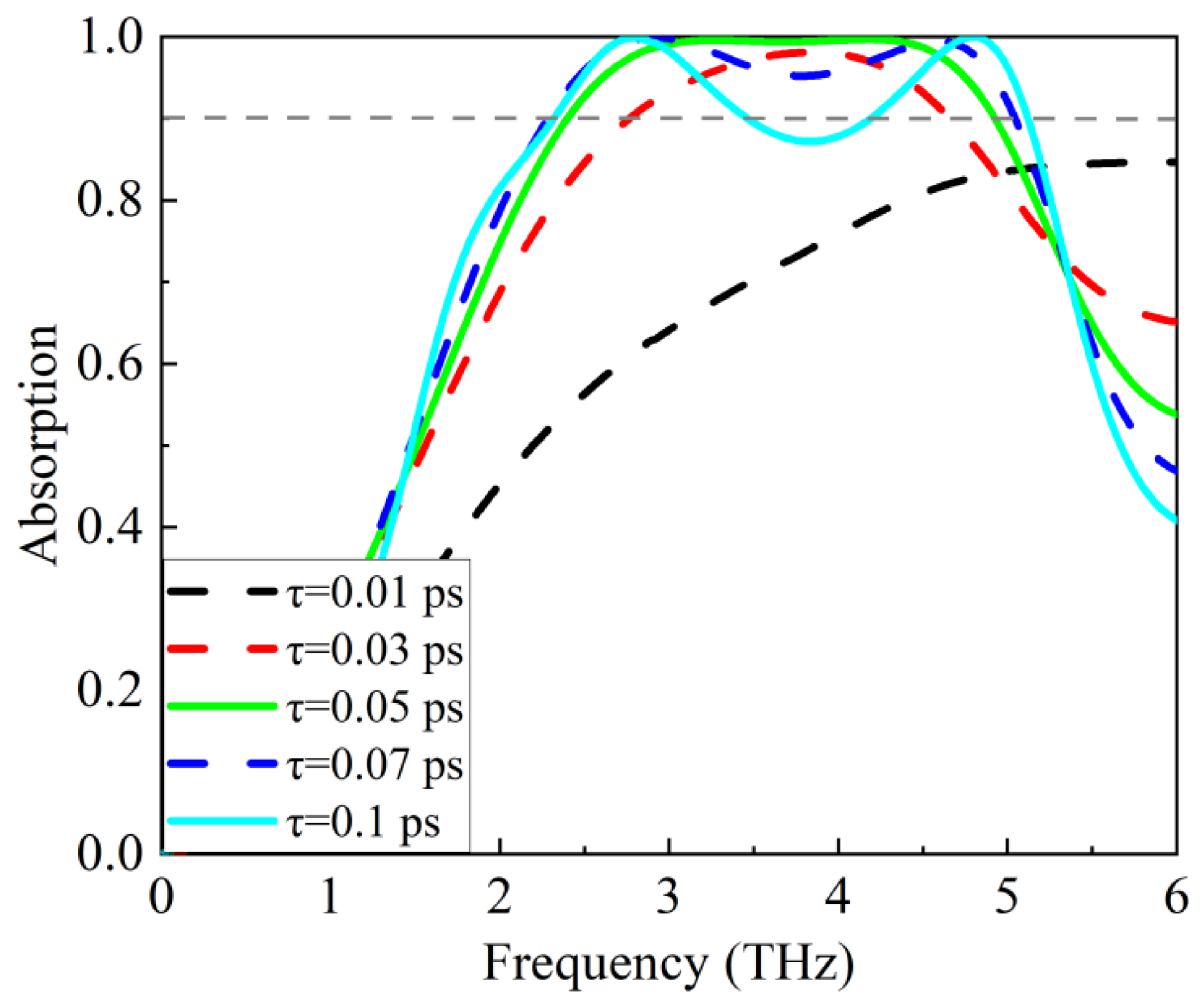
Absorption curves with different τ values when $E_{f}$ = 0.9 eV.

Figure 13 depicts the absorption spectra of the THz absorber with $E_{f}$ fixed at 0.9 eV, as a function of *τ* varying in the range of 0.01–0.1 ps. It can be clearly observed from the figure that the absorption performance of the THz absorber is closely associated with *τ*. Specifically, when *τ* increases from 0.01 ps to 0.05 ps, the absorption intensity is significantly enhanced, and the absorption curve exhibits a trend of transitioning from a low-efficiency flat profile, gradually concentrating, to a high-efficiency flat profile. As *τ* further increases from 0.05 ps to 0.1 ps, the absorption curve transforms from the previous high-efficiency flat shape into two narrowband, high-efficiency absorption peaks.

To further verify the effectiveness and novelty of this work, a comprehensive comparison with recently reported graphene-based THz absorbers [15,22,23,24,25] is presented in Table 1, covering four core dimensions (absorption bandwidth, absorption efficiency, tunability and fabrication cost). As summarized in Table 1, the proposed absorber exhibits better comprehensive performance. First, in terms of absorption performance, it achieves a 2.5 THz 90% absorption bandwidth and a peak absorptivity of 99.8%, along with a 1.4 THz near-perfect absorption band (≥99%), which is superior to most of the compared works. Second, different from previous designs [15,22,23] that only support broadband absorption, this work realizes switching between narrowband and broadband absorption. Finally, in terms of fabrication cost, the proposed absorber adopts low-cost tungsten as the reflective layer instead of expensive noble metals (e.g., Au) widely used in reported works, which reduces the material cost.

## 5. Conclusions

To address the demand for tunable broadband absorbers in THz technologies, this work proposes a graphene-based THz absorber with a sandwich structure, consisting of a bottom tungsten reflective layer, a middle PI dielectric layer, and a top patterned graphene absorption layer. FEM simulations are carried out to explore the combined effects of $E_{f}$ and *τ* on absorption performance across the 0–6 THz range. It is revealed that increasing $E_{f}$ from 0.1 eV to 0.9 eV leads to a significant broadening of the absorption band with absorptivity exceeding 90%. At *τ* = 0.1 ps, the proposed THz absorber exhibits two narrowband absorptions with a maximum absorptivity of 99.8%. In contrast, at *τ* = 0.05 ps, the absorber exhibits a broadband response of 2.5 THz (2.4–4.9 THz), including a 1.4 THz near-perfect absorption bandwidth with absorptivity exceeding 99%, thus enabling switchable broadband and narrowband operation. Different from conventional designs using expensive noble metals, the adoption of tungsten greatly cuts fabrication costs while maintaining excellent reflection performance. The proposed device also features favorable polarization insensitivity and angular stability. This work provides practical design references for low-cost switchable THz absorbers.

## Figures and Tables

**Table 1:** Performance comparison with recently reported THz absorbers.

Reference	Absorption Bandwidth (≥90%)	Absorption Rate	Tunability	Fabrication Cost
This work	2.5 THz	99.80%	Narrowband–Broadband Switchable	Low
Ref. [15]	1.96 THz	99.12%	Broadband only	High
Ref. [22]	2 THz	99.70%	Broadband only	High
Ref. [23]	2.26 THz	98.00%	Broadband only	Medium
Ref. [24]	1.8 THz	>90%	Narrowband–Broadband Switchable	Medium
Ref. [25]	1.34 THz	>90%	Narrowband–Broadband Switchable	Medium

## Data Availability

The datasets used and/or analyzed during the current study are available from the corresponding author on reasonable request.
